# Sanitation-related violence against women in informal settlements in Kenya: a quantitative analysis

**DOI:** 10.3389/fpubh.2023.1191101

**Published:** 2023-09-29

**Authors:** Samantha C. Winter, Laura Johnson, Millicent N. Dzombo

**Affiliations:** ^1^School of Social Work, Columbia University, New York, NY, United States; ^2^School of Social Work, Temple University, Philadelphia, PA, United States; ^3^Columbia Global Center, Columbia University, Nairobi, Kenya

**Keywords:** violence against women, Kenya, slums (informal settlements), urban, Africa

## Abstract

**Introduction:**

Approximately 3.6 billion people around the world do not have access to safe sanitation options. Those lacking access are not only at risk of diarrheal disease, other tropical diseases, and parasitic infections, they are at greater risk of experiencing violence, particularly women and girls. The burden of lack of access to safe sanitation is disproportionately experienced by women in informal settlements in lower- and middle-income countries, where violence rates tend to be higher and access to sanitation lower. Women lacking access to safe toilets often have to walk long distances to access a facility or open site or use shared toilet facilities, which increase their vulnerability to violence.

**Methods:**

We explore the prevalence and multilevel factors associated with women's experiences, observations, and exposure to stories about past-year sanitation-related violence in a probability sample of 550 women in a large informal settlement in Nairobi, Kenya using chi-square tests and multivariate logistic regressions.

**Results:**

Findings suggest that social/community engagement and social/cultural beliefs are important considerations for hearing about and observing sanitation-related violence, but less so experiences of sanitation-related violence. Alternatively, individual-level and technological factors may be critical factors in actual experiences of violence.

**Discussion:**

Sanitation-related violence and creating an environment of safety in which women can take care of their sanitation-related needs in ways that also protect them, their families, and their communities is critical for meeting sanitation-related development agendas and goals such as Sustainable Development Goal 6.2 to achieve access to adequate and equitable sanitation and hygiene for all by 2030.

## Introduction

Lack of access to safe sanitation is associated with serious health risks, and disproportionately exposes women and girls to violence (1–11). Guided by development goals such as Sustainable Development Goal 6.2, which calls for “achieving access to adequate and equitable sanitation and hygiene for all,” there have been many efforts over the last three decades to reduce the number of people living without sanitation access. However, approximately 46% of people (about 3.6 billion) around the world still do not have adequate access to safely-managed sanitation services (12). Those lacking safely-managed toilets—defined as sanitation facilities which hygienically separate human excreta from human contact; are not shared with other households; and have a process in place for treating and disposing of excreta *in-situ*, temporarily storing and emptying and treating excreta offsite, or transporting excreta through a sewer with wastewater to be treated off-site—rely on shared toilet facilities, basic toilets or latrines, poorly-constructed latrines, buckets, or open defecation (13).

While lack of access to safe sanitation is a global public health issue, the burden of sanitation deficiencies falls disproportionately on vulnerable and under-resourced populations and communities. For example, access to safe sanitation remains relatively low in sub-Saharan Africa, particularly in rural areas (13). Although it is difficult to analyze sanitation access trends in informal settlements because the populations and boundaries of these communities shift and are difficult to define, individual studies suggest that access to and utilization of sanitation in settlements is limited, especially for women (14–18). There are a number of studies that suggest women who lack access to adequate sanitation are at greater risk of experiencing violence (1, 3–11). Literature focused on the gendered aspects of sanitation highlight harmful social norms as the key factors in women's disproportionate vulnerability to sanitation-related violence. For example, while it is socially acceptable for men in many societies to relieve themselves in relatively public spaces, social attributions of shame around women's sanitation practices may expose women to gender-based violence as they try to find and utilize adequately private places to meet their sanitation needs (2). Alternatively, they may be at risk for harassment when they do not conform to expected practices. Social taboos around menstruation and the associated increased need for privacy add to women's vulnerability to sanitation-related violence (2). Despite a growing body of research acknowledging women's disproportionate vulnerability to sanitation-related violence, there are few studies that have quantitatively explored this phenomenon in informal settlements in sub-Saharan Africa (1). The purpose of this study was to explore the prevalence and multilevel factors associated with women's sanitation-related violence, which is being defined, for the purposes of this study, as any verbal, sexual, or physical harassment that specifically takes place while a woman is walking to/from or utilizing a toilet or other site/method for sanitation (e.g., open field, drainage, plastic/paper bag or bucket).

### Sanitation in informal settlements

Approximately one billion people, globally, reside in informal settlements, including close to 60% of the urban population in Africa (19). While there is no official definition of an informal settlement beyond unplanned settlements not authorized by the State (20), scholars and development entities have created practical definitions to help demarcate these settlements from other urban neighborhoods and populations, and all of these definitions incorporate lack of access to sanitation as a criterion. The most widely-used definition, for example, defines informal settlements (slums) as areas that meet any one of five conditions: lack of clean water, lack of sanitation, non-durable construction, overcrowding, and insecure tenure [(21), p. 19].

Lack of access to adequate and safe sanitation has been associated with a number of poor health outcomes including diarrheal disease—one of the leading causes of death in children under five—as well as other tropical diseases such as cholera and typhoid and parasitic infections (22–24). The problem of lack of adequate sanitation can be even more critical for people living in informal settlements, where sanitation coverage rates tend to be lower [e.g., 32% of the urban poor in sub-Saharan Africa use improved sanitation compared to 85% of wealthy urban dwellers (25)], and, due to intimately shared social and physical environments, neighborhood-level health risks such as the rapid spread of communicable diseases tend to be higher (26).

In Nairobi, Kenya, the site for this study, over 60% of the 3 million residents live in informal settlements (27), and only about 24% of households in these communities have access to improved sanitation (28). Research in Nairobi suggests that access to adequate sanitation in informal settlements in a persistent challenge (29), particularly for women (18).

### Sanitation-related violence

In addition to the health issues that lack of access to improved sanitation poses to individuals and neighborhoods, especially those located in informal settlements, literature suggests women may be at greater risk of experiencing violence when utilizing inadequate toilets or sanitation sites/methods (1, 2, 4–7, 10, 11, 30). Findings from these studies suggest that lack of access to adequate sanitation may increase women's and girls' vulnerability to sexual harassment, rape, or other forms of violence (1, 2, 30, 31). In areas with limited or no toilet facilities, women may wait until nightfall and/or travel long distances from their homes to find a private place to relieve themselves, which can increase their vulnerability to violence (5, 7, 32). In informal settlement communities, in particular, women who rely on shared toilet facilities as compared to private, indoor facilities have higher odds of experiencing non-partner violence (1). Studies have also suggested that characteristics of toilets, such as lack of privacy, may increase women's risk and/or fears of sexual violence (2, 33). In response to experiences or fears of violence, women may adopt sanitation-related coping strategies they perceive to be safer, including reverting to the use of buckets or plastic bags in their homes (18, 31), restricting food and liquid intake, and/or withholding urination or defecation (7); however, these coping behaviors can also have health implications for women such as increased risk of infections and toxic shock syndrome (34–37).

Despite the many studies and reports that have illustrated or discussed an association between inadequate sanitation and women's experiences and/or fear of violence, mostly non-partner violence (NPV), there are no quantitative analyses to date documenting prevalence and/or factors that may increase or decrease risk of sanitation-related violence or fear of this type of violence beyond type of toilet/sanitation-management method, especially in informal settlements. The purpose of this study, therefore, was to help fill this gap by exploring the prevalence and multilevel factors associated with women's experiences, observations, and exposure to stories about past-year sanitation-related violence in a large informal settlement in Nairobi, Kenya.

## Materials and methods

### Data

Data for these analyses were collected as part of a quantitative study focused on women's access to and experiences of space and services, e.g., water and sanitation, in Mathare Valley Informal Settlement (Mathare) in Nairobi, Kenya in 2016. Women were asked about characteristics and experiences getting to and using services like water and sanitation in their communities as well as their general perceptions of safety, crime, insecurity, and cohesion in their communities and neighborhoods. The Mathare informal settlement is home to ~200,000 residents, and is one of the most densely populated areas in Kenya (38). Mathare is divided into villages. While the boundaries of these villages are contested and would likely be missed by outsiders, the residents of these settlements know the boundaries well. Data for this study were collected in all 11 major villages that women living in Mathare identified as comprising the informal settlement.

### Sample

The present analyses are based on cross-sectional data from 550 household-level surveys collected from 50 women in each of the 11 villages. Eleven women from Mathare (one from each village) were trained in research ethics and quantitative research methods and they administered the surveys. Households for the survey were randomly selected within each village using the fishnet and point sampling tools in ArcMAP (39). This technique has been employed to assist in random household selection in other studies conducted in informal settlements (40, 41). Some of the random points generated in ArcMAP were on or near the boundary of the villages. These cases were reviewed after the collection of the survey and the research team decided to which village the survey should be assigned. These decisions were made with the 11 women on the data collection team.

Women chosen to collect community data were involved in previous research studies carried out by the research team, and were selected for this role because of their enthusiasm for the research topic and their extensive lived-experience as informal leaders, volunteers, and/or workers in health and women's initiatives in their respective villages. They were residents of each of the 11 villages in Mathare. Though there were no educational requirements for the position, women had to be able to communicate and read and write in English and Swahili at a level sufficient to understand and clearly guide participants through the informed consent process and the survey instrument, follow survey instructions and skip patterns on pencil-and-paper surveys, and fill in responses correctly on behalf of participants. In order to prepare them for these tasks, community members received in-depth didactic and interactive trainings in quantitative research methods, study-specific protocols and instruments, and methods for researching violence against women and sensitive topics (42). The research team observed mock informed consent and survey sessions for all community data collection and reviewed all completed surveys for compliance, quality, and completeness within 48 hours of survey administration. Feedback was given to team members after both mock sessions and survey review.

In order to be eligible to participate in the survey, women had to be at least 18 years old and have lived in the informal settlement for at least 6 months (i.e., were not visitors). If more than one woman in the household met the eligibility criteria to participate, a Kish grid was used to randomly select one participant to invite into the study (43).

### Data collection

Quantitative surveys were administered to one woman in each household. All participants were taken through an informed consent process by a member of the data collection team, and then asked to provide oral consent if they wished to be part of the study. Oral consent was deemed appropriate by the reviewing ethics committee because written consent would have been the only identifying data linking otherwise anonymous surveys to participants (45 CFR § 46.117). If participants consented, the member of the data collection team administering the survey signed a document affirming consent. Survey questions were read aloud to participants in English or Swahili, depending on participant preference, and filled in on paper surveys. Surveys took approximately 40–60 min to complete.

### Measures

#### Primary outcome variables: sanitation-related violence

Sanitation-related violence was measured using a series of 18 items that examined three forms of sanitation-related violence exposure: experienced, observed, and heard about violence. Participants in the study were asked a set of six separate questions about experiencing, observing, and hearing about sanitation-related violence in the past 12 months, i.e., “In the past 12 months, have you been/have you observed another woman being/have you heard of another woman being: (1) physically attacked *while walking to or from* a toilet/sanitation site, (2) physically attacked *while using* a toilet/sanitation site, (3) sexually harassed/raped *while walking to or from* a toilet/sanitation site, (4) sexually harassed/raped *while using* a toilet/sanitation site, (5) verbally harassed *while walking to or from* a toilet/sanitation site, and (6) verbally harassed *while using* a toilet/sanitation site. A dichotomous yes/no variable was created for each of the three outcome variables (experienced, observed, heard about violence) if a woman reported “yes” to any of the six questions related to that outcome.

#### Predictor variables

All potential predictor variables in this study were chosen based on factors that have been associated with women's risk or experiences of sanitation-related violence in previous literature. For ease of interpretation, variables are organized into ecological levels—individual, interpersonal/household, technological, community/neighborhood and social/cultural.

##### Individual factors

Socio-demographic variables, such as respondent's education level, age, employment status, and a woman's self-reported health status were included. In addition, responses from questions to assess women's sense of safety in relation to sanitation (44) were used to capture information about women's safety and fear of victimization in the community and/or related to her primary toilets/sanitation sites. Responses to these questions were used to develop dichotomous variables that reflected whether or not women: (1) feel it is safe to walk alone in the neighborhood (during the day/at night), (2) fear victimization (during the day/at night), and (3) feel their current toilet/sanitation site is secure (during the day/at night). A three-level fear of crime in the neighborhood Likert-scale variable was also included (range 0 = not at all worried, 1 = a little worried, 2 = very worried). Variables capturing whether a woman was satisfied with her toilet/method of disposal, whether she felt she had privacy when using her toilet/method of disposal, and whether she felt embarrassed using her toilet/method of disposal were included.

##### Interpersonal/household factors

Household income, relationship status, residential stability (how long a woman had resided in her current household), number of children, and the gender of the head of household were included. A dichotomous variable reflecting whether or not a respondent participates in at least one group (church, social, microfinance, etc.) in the community was also included.

##### Technological factors

Women were asked questions about their methods for management of urine and feces during the day and night because most women in settlements use more than one method and up to four different methods during a 24-h period (18). For the purposes of these analyses, we focused on daytime and nighttime methods. Variables to indicate whether a women's toilet(s)/sanitation site(s) had structural characteristics to help ensure safety and privacy, i.e., a door on the toilet stall, a lock on the door, separate gender stalls, and lights, were included. The following technological variables were also included: whether a respondents' daytime toilet/site is closed at night; whether or not a respondent regularly waits in line to use their toilet(s)/site(s); whether the respondents' toilet(s)/site(s) are sometimes not accessible; whether there is a fee to use the toilet(s); the walking time to reach toilet(s)/site(s); whether or not the respondents' toilet(s)/site(s) are shared with other people; whether or not the toilet(s)/site(s) can be used by any member of the public; and whether or not respondents' use bags or buckets in the home for their disposal method(s). Variables were also included to capture information about characteristics of respondents' toilet(s)/site(s) such as whether the toilet(s)/site(s) are clean; whether respondents' toilet(s)/site(s) have a bin for disposing of feminine hygiene products; and whether a respondent's toilet(s)/site(s) have running water.

##### Community factors

Since many women in informal settlements rely on a water source located somewhere in the community outside their homes, housing plots or buildings, a variable for location/type of primary water source was included in the analyses (4 categories: 1. Tap or well inside the house or housing plot, 2. Private tap or well outside the housing plot or building, 3. Public tap or well outside the housing plot or building, 4. Water tanker or vendor). Residential stability (how long a woman has resided in her community) as well as a scale for neighborhood cohesion (45) and a scale for neighborhood disorganization (46) were also included.

##### Societal/cultural factors

Finally, four binary variables: (1) my culture has rules about the disposal of urine/feces, (2) my culture has rules about hygiene, (3) my religion has rules about the disposal of urine/feces, and (4) my religion has rules about hygiene, were included in the models.

### Analysis strategy

The analysis for this study utilized responses from all 550 household surveys. There were minimal missing responses (less than one percent) on all variables included in this analysis. Random, single-response imputation was used to fill in the values for any missing responses using the user-written program hotdeckvar (47) in Stata v.15 (48). Analyses were conducted in Stata statistical software [version 15; StataCorp (48)].

Study analyses were guided by the following research questions: (R1) What are the multilevel factors associated with hearing about another woman's sanitation-related violence? (R2) What are the multilevel factors associated with observing another woman's sanitation-related violence? (R3) What are the multilevel factors associated with experiencing sanitation-related violence?

Descriptive statistics were calculated to provide information about the study sample and the prevalence rates of the three sanitation-related violence outcomes: heard about sanitation-related violence, observed sanitation-related violence, and experienced sanitation-related violence. Variable characteristics, including frequencies for categorical variables and means and standard deviations for continuous variables are presented in Tables 1–3. The first step in exploring correlates of the three sanitation-related violence outcomes and answering research questions 1–3 was to run Pearson's Chi-Squared tests (for categorical variables) and bivariate logistic regressions (for continuous variables). Subsequently, multivariate logistic regressions were run to explore correlates of (R1) hearing about sanitation-related violence (R2), observing sanitation-related violence, and (R3) experiencing sanitation-related violence while controlling for covariates. We followed the guidance of Hosmer et al. (49) and Bursac et al. (50) for purposeful selection of covariates to build our logistic regression models, which involves several steps: (1) use univariate analyses to explore the unadjusted association between each independent variable and the outcome; (2) variables with a *p* < 0.25 from step 1 are included in a multivariable analysis; (3) variables with a *p* < 0.1 are eliminated one-by-one and a new model is fit after each elimination unless removal of the variable causes more than a 20% change in any of the other parameters in the model. If the removal of the variable causes more than a 20% change in any of the other parameters, it is considered a confounder and kept in the model. Each new model is compared to the previous model using a partial likelihood ratio test to make sure the new model fits as well as the previous model; (4) after the model contains only significant covariates and confounders, all variables not selected for the original multivariable model is added back into the final model from step 4 one at a time. Those that are significant at the 0.1 level are kept in the model. The model is iteratively reduced as in step 3 for each of the variables that were added back in. After the 4th step, you are left with the final, parsimonious model. We ran this process (steps 1–4) for each violence outcome, separately. We also compared the Akaike Information Criterion (AIC) and Bayes Information Criterion (BIC) for each model to ensure that the fit was better than the multivariable model in step 2 (51).

**Table 1:** Proportions and bivariate associations with past-year experiences of sanitation-related violence.

	**% [mean (std)]**	**No viol**	**Yes viol**	**Chi-squared**
**Dependent variables**				
Experienced sanitation-related violence in past 12 months	12.73			–
Observed sanitation-related violence in past 12 months	13.27	60.27	39.73	χ^2^ (1, *N =* 549) = 12.223, *p =* 0.006
Heard about sanitation-related violence in past 12 months	23.64	70.00	30.00	χ^2^ (1, *N =* 549) = 6.276, *p =* 0.031
**Individual-level factors**				
Respondent age (continuous)	32.21 (0.786)			OR = 1.04 (95% CI: 1.015, 1.066), *p =* 0.005
Respondent education level				χ^2^ (3, *N =* 547) = 0.965, *p =* 0.412
Less than primary	18.55	91.18	8.82	
Completed primary, no secondary	24.73	86.03	13.97	
Some secondary, but did not complete	22.18	85.25	14.75	
Completed secondary	34.55	87.37	12.63	
Respondent employed	45.45	83.60	16.40	χ^2^ (1, *N =* 549) = 5.427, *p =* 0.042
Respondent reports good/excellent health	57.82	86.48	13.52	χ^2^ (1, *N =* 549) = 0.108, *p =* 0.750
Respondent believes it is safe to walk alone in the neighborhood during the day	97.82	87.36	12.64	χ^2^ (1, *N =* 549) = 0.061, *p =* 0.810
Respondent believes it is safe to walk alone in the neighborhood at night	19.64	90.74	9.26	χ^2^ (1, *N =* 549) = 0.0628, *p =* 0.446
Respondent fears victimization during the day	0.91	60.00	40.00	χ^2^ (1, *N =* 549) = 1.421, *p =* 0.261
Respondent fears victimization at night	75.45	86.99	13.01	χ^2^ (1, *N =* 549) = 0.043, *p =* 0.839
Respondent identified insecurity as an issue with their toilet during the day	5.27	79.31	20.69	χ^2^ (1, *N =* 549) = 1.545, *p =* 0.242
Respondent identified insecurity as an issue with their toilet during the night	34.36	83.07	16.93	χ^2^ (1, *N =* 549) = 1.644, *p =* 0.229
Worried about crime in neighborhood				χ^2^ (2, *N =* 548) = 3.663, *p =* 0.058
Not at all worried	11.45	95.24	4.76	
A little worried	22.00	93.39	6.61	
Very worried	66.55	83.88	16.12	
Respondent is satisfied with their toilet during the day	32.91	87.29	12.71	χ^2^ (1, *N =* 549) = 0.000, *p =* 0.994
Respondent is satisfied with their toilet at night	50.91	86.43	13.57	χ^2^ (1, *N =* 549) = 0.136, *p =* 0.720
Respondent reports privacy in toilet during the day	67.09	90.79	9.21	χ^2^ (1, *N =* 549) = 3.997, *p =* 0.034
Respondent reports privacy in toilet at night	41.09	92.48	7.52	χ^2^ (1, *N =* 549) = 4.673, *p =* 0.056
Respondent is embarrassed to use toilet during the day	34.55	81.05	18.95	χ^2^ (1, *N =* 549) = 3.944, *p =* 0.075
Respondent is embarrassed to use toilet during the night	53.27	84.64	15.36	χ^2^ (1, *N =* 549) = 1.480, *p =* 0.252
**Interpersonal/household-level factors**				
Household monthly income (KES)				χ^2^ (2, *N =* 548) = 0.649, *p =* 0.520
< 5,000	25.09	85.51	14.49	
KES 5,000–10,000	50.55	85.97	14.03	
>10,000	24.36	91.79	8.21	
Relationship status				χ^2^ (3, *N =* 547) = 0.410, *p =* 0.683
Married	54.73	87.38	12.62	
Living with partner, but not married	2.00	90.91	9.09	
Regular partner, living apart	6.36	91.30	8.70	
Single	34.91	85.94	14.06	
Residential stability in household				χ^2^ (4, *N =* 546) = 0.8621, *p =* 0.4494
< 1 year	13.82	86.84	13.16	
Between 1 and 4 years	44.91	87.45	12.55	
Between 5 and 9 years	24.36	91.04	8.96	
Between 10 and 19 years	8.00	84.09	15.91	
>20 years	8.91	79.59	20.41	
Respondent has children	81.64	87.08	12.92	χ^2^ (1, *N =* 549) = 0.086, *p =* 0.775
Respondent number of children (continuous)	2.21 (0.067)			OR = 1.14 (95% CI: 0.965, 1.345), *p* = 0.110
Head of household is male	57.45	87.66	12.34	χ^2^ (1, *N =* 549) = 0.419, *p =* 0.532
Respondent participates in one or more groups	58.55	85.09	14.91	χ^2^ (1, *N =* 549) = 0.8230, *p =* 0.386
**Technological-level factors**				
Respondent's daytime toilet has doors	85.09	87.82	12.18	χ^2^ (1, *N =* 549) = 0.412, *p =* 0.5353
Respondent's nighttime toilet has doors	35.27	90.21	9.79	χ^2^ (1, *N =* 549) = 1.920, *p =* 0.196
Respondent's daytime toilet has locks on doors	74.91	88.83	11.17	χ^2^ (1, *N =* 549) = 0.953, *p =* 0.352
Respondent's nighttime toilet has locks on doors	32.91	92.27	7.73	χ^2^ (1, *N =* 549) = 6.624, *p =* 0.028
Respondent's daytime toilet has separate gender stalls	27.45	90.07	9.93	χ^2^ (1, *N =* 549) = 0.802, *p =* 0.392
Respondent's nighttime toilet has separate gender stalls	6.73	83.27	16.73	χ^2^ (1, *N =* 549) = 1.212, *p =* 0.297
Respondent's daytime toilet has lights	30.00	90.91	9.09	χ^2^ (1, *N =* 549) = 1.006, *p =* 0.340
Respondent's nighttime toilet has lights	10.36	89.47	10.53	χ^2^ (1, *N =* 549) = 0.153, *p =* 0.704
Respondent's daytime toilet is closed at night	42.91	86.86	13.14	χ^2^ (1, *N =* 549) = 0.044, *p =* 0.838
Respondent has to queue to use toilet during the day	63.64	86.00	14.00	χ^2^ (1, *N =* 549) = 0.716, *p =* 0.417
Respondent has to queue to use toilet at night	22.91	86.51	13.49	χ^2^ (1, *N =* 549) = 0.119, *p =* 0.737
Respondent's toilet is sometimes not accessible during the day	77.64	86.89	13.11	χ^2^ (1, *N =* 549) = 0.137, *p =* 0.719
Respondent's toilet is sometimes not accessible at night	31.45	89.02	10.98	χ^2^ (1, *N =* 549) = 0.635, *p =* 0.444
Respondent pays fee to access daytime toilet	42.91	85.59	14.41	χ^2^ (1, *N =* 549) = 0.787, *p =* 0.396
Respondent pays fee to access nighttime toilet	3.82	90.48	9.52	χ^2^ (1, *N =* 549) = 0.202, *p =* 0.663
Walk time to reach daytime toilet				χ^2^ (4, *N =* 546) = 0.495, *p =* 0.590
Does not walk	31.09	90.06	9.94	
< 1 min	15.64	87.21	12.79	
Between 1 and 2 min	19.45	83.18	16.82	
Between 3 and 4 min	14.73	85.19	14.81	
Five or more min	19.09	88.57	11.43	
Walk time to reach nighttime toilet				χ^2^ (4, *N =* 546) = 0.926, *p =* 0.439
Does not walk	66.18	85.99	14.01	
< 1 min	16.18	88.76	11.24	
Between 1 and 2 min	6.73	94.59	5.41	
Between 3 and 4 min	5.27	86.21	13.79	
Five or more min	5.64	90.32	9.68	
Respondent uses shared toilet during the day	79.82	87.02	12.98	χ^2^ (1, *N =* 549) = 0.065, *p =* 0.804
Respondent uses shared toilet at night	32.18	89.83	10.17	χ^2^ (1, *N =* 549) = 2.171, *p =* 0.171
Respondent uses public toilet during the day	50.36	85.92	14.08	χ^2^ (1, *N =* 549) = 0.972, *p =* 0.347
Respondent uses public toilet at night	7.45	85.37	14.63	χ^2^ (1, *N =* 549) = 0.100, *p =* 0.758
Respondent uses bags/buckets during the day	29.64	81.60	18.40	χ^2^ (1, *N =* 549) = 3.044, *p =* 0.112
Respondent uses bags/buckets at night	68.73	85.45	14.55	χ^2^ (1, *N =* 549) = 2.288, *p =* 0.161
Respondent's daytime toilet is clean	61.64	92.63	7.37	χ^2^ (1, *N =* 549) = 17.38, *p =* 0.002
Respondent's nighttime toilet is clean	29.09	93.12	6.88	χ^2^ (1, *N =* 549) = 7.215, *p =* 0.023
Respondent's daytime toilet has running water	32.12	88.64	11.36	χ^2^ (1, *N =* 549) = 0.581, *p =* 0.464
Respondent's nighttime toilet has running water	10.18	87.50	12.50	χ^2^ (1, *N =* 549) = 0.003, *p =* 0.959
Respondent's daytime toilet has a bin for pads	18.91	91.35	8.65	χ^2^ (1, *N =* 549) = 0.910, *p =* 0.363
Respondent's nighttime toilet has a bin for pads	7.64	90.48	9.52	χ^2^ (1, *N =* 549) = 0.308, *p =* 0.591
**Community/neighborhood-level factors**				
Primary water source				χ^2^ (3, *N =* 547) = 1.089, *p =* 0.352
Tap/well inside house/plot/building	13.45	85.14	14.86	
Tap/well outside house/plot/building	16.18	82.02	17.98	
Public tap/well	56.73	88.14	11.86	
Tanker/vendor	13.64	92.00	8.00	
Residential stability in community				χ^2^ (4, *N =* 546) = 0.357, *p =* 0.810
< 1 year	5.27	89.66	10.34	
Between 1 and 4 years	24.91	88.32	11.68	
Between 5 and 9 years	24.00	87.12	12.88	
Between 10 and 19 years	2.00	89.09	10.91	
>20 years	25.82	84.51	15.49	
Neighborhood cohesion (mean)	0.78 (0.030)			OR = 0.17 (95% CI: 0.033,0.839), *p* = 0.033
Neighborhood cohesion (sum)	14.10 (0.540)			OR = 0.90 (95% CI: 0.827, 0.990), *p* = 0.033
Neighborhood disorganization (mean)	2.28 (0.094)			OR = 3.08 (95% CI: 0.775, 12.251), *p* = 0.099
Neighborhood disorganization (sum)	36.44 (1.503)			OR = 1.07 (95% CI: 0.984, 1.169), *p* = 0.099
**Social/cultural-level factors**				
Respondent's religion has rules about disposal of feces	8.18	86.67	13.33	χ^2^ (1, *N =* 549) = 0.014, *p =* 0.909
Respondent's religion has rules about hygiene	10.55	82.76	17.24	χ^2^ (1, *N =* 549) = 0.783, *p =* 0.397
Respondent's culture has rules about disposal of feces	19.82	88.07	11.93	χ^2^ (1, *N =* 549) = 0.099, *p =* 0.759
Respondent's culture has rules about hygiene	18.73	87.38	12.62	χ^2^ (1, *N =* 549) = 0.002, *p =* 0.969

**Table 2:** Proportions and bivariate associations with past-year observations of sanitation-related violence.

	**% [mean (std)]**	**No viol**	**Yes viol**	**Chi-squared**
**Dependent variable**				
Observed sanitation-related violence in past 12 months	13.27			–
**Individual-level factors**				
Respondent age (continuous)	32.21 (0.786)			OR = 1.05 (95% CI: 1.020, 1.088), *p* = 0.005
Respondent education level				χ^2^ (3, *N =* 547) = 1.070, *p =* 0.364
Less than primary	18.55	85.29	14.71	
Completed primary, no secondary	24.73	83.82	16.18	
Some secondary, but did not complete	22.18	90.98	9.02	
Completed secondary	34.55	86.84	13.16	
Respondent employed	45.45	87.2	12.8	χ^2^ (1, *N =* 549) = 0.152, *p =* 0.705
Respondent reports good/excellent health	57.82	86.48	13.52	χ^2^ (1, *N =* 549) = 0.017, *p =* 0.900
Respondent believes it is safe to walk alone in the neighborhood during the day	97.82	86.99	13.01	χ^2^ (1, *N =* 549) = 1.6117, *p =* 0.233
Respondent believes it is safe to walk alone in the neighborhood at night	19.64	95.37	4.63	χ^2^ (1, *N =* 549) = 12.12, *p =* 0.006
Respondent fears victimization during the day	0.91	80	20	χ^2^ (1, *N =* 549) = 16.93, *p =* 0.689
Respondent fears victimization at night	75.45	84.1	15.9	χ^2^ (1, *N =* 549) = 19.36, *p =* 0.001
Respondent identified insecurity as an issue with their toilet during the day	5.27	86.21	13.79	χ^2^ (1, *N =* 549) = 0.03, *p =* 0.878
Respondent identified insecurity as an issue with their toilet during the night	34.36	86.77	13.23	χ^2^ (1, *N =* 549) = 0.00, *p =* 0.990
Worried about crime in neighborhood				χ^2^ (2, *N =* 548) = 1.42, *p =* 0.263
Not at all worried	11.45	95.24	4.76	
A little worried	22.00	89.26	10.74	
Very worried	66.55	84.43	15.57	
Respondent is satisfied with their toilet during the day	32.91	86.19	13.81	χ^2^ (1, *N =* 549) = 0.06, *p =* 0.807
Respondent is satisfied with their toilet at night	50.91	84.29	15.71	χ^2^ (1, *N =* 549) = 2.34, *p =* 0.157
Respondent reports privacy in toilet during the day	67.09	89.43	10.57	χ^2^ (1, *N =* 549) = 2.85, *p =* 0.123
Respondent reports privacy in toilet at night	41.09	90.71	9.29	χ^2^ (1, *N =* 549) = 3.33, *p =* 0.098
Respondent is embarrassed to use toilet during the day	34.55	80	20	χ^2^ (1, *N =* 549) = 6.90, *p =* 0.025
Respondent is embarrassed to use toilet during the night	53.27	83.62	16.38	χ^2^ (1, *N =* 549) = 2.61, *p =* 0.137
**Interpersonal/household-level factors**				
Household monthly income (KES)				χ^2^ (2, *N =* 548) = 0.2033, *p =* 0.767
< 5,000	25.09	86.23	13.77	
KES 5,000–10,000	50.55	85.61	14.39	
>10,000	24.36	89.55	10.45	
Relationship status				χ^2^ (3, *N =* 547) = 0.244, *p =* 0.799
Married	54.73	86.05	13.95	
Living with partner, but not married	2.00	90.91	9.09	
Regular partner, living apart	6.36	89.13	10.87	
Single	34.91	86.98	13.02	
Residential stability in household				χ^2^ (4, *N =* 546) = 0.8895, *p =* 0.4585
< 1 year	13.82	88.16	11.84	
Between 1 and 4 years	44.91	87.04	12.96	
Between 5 and 9 years	24.36	88.81	11.19	
Between 10 and 19 years	8.00	77.27	22.73	
>20 years	8.91	85.71	14.29	
Respondent has children	81.64	85.75	14.25	χ^2^ (1, *N =* 549) = 6.877, *p =* 0.026
Respondent number of children (continuous)	2.21 (0.067)		100	OR = 1.21 (95% CI: 1.080, 1.354), *p* = 0.004
Head of household is male	57.45	85.76	14.24	χ^2^ (1, *N =* 549) = 1.295, *p =* 0.282
Respondent participates in one or more groups	58.55	83.54	16.46	χ^2^ (1, *N =* 549) = 2.362, *p =* 0.155
**Technological-level factors**				
Respondent's daytime toilet has doors	85.09	85.9	14.1	χ^2^ (1, *N =* 549) = 1.54, *p =* 0.243
Respondent's nighttime toilet has doors	35.27	89.18	10.82	χ^2^ (1, *N =* 549) = 2.33, *p =* 0.158
Respondent's daytime toilet has locks on doors	74.91	87.14	12.86	χ^2^ (1, *N =* 549) = 0.09, *p =* 0.767
Respondent's nighttime toilet has locks on doors	32.91	90.61	9.39	χ^2^ (1, *N =* 549) = 0.09, *p =* 0.767
Respondent's daytime toilet has separate gender stalls	27.45	88.08	11.92	χ^2^ (1, *N =* 549) = 0.13, *p =* 0.729
Respondent's nighttime toilet has separate gender stalls	6.73	89.19	10.81	χ^2^ (1, *N =* 549) = 0.22, *p =* 0.651
Respondent's daytime toilet has lights	30.00	89.7	10.3	χ^2^ (1, *N =* 549) = 0.58, *p =* 0.465
Respondent's nighttime toilet has lights	10.36	91.23	8.77	χ^2^ (1, *N =* 549) = 0.94, *p =* 0.356
Respondent's daytime toilet is closed at night	42.91	83.05	16.95	χ^2^ (1, *N =* 549) = 4.53, *p =* 0.0591
Respondent has to queue to use toilet during the day	63.64	84.29	15.71	χ^2^ (1, *N =* 549) = 10.25, *p =* 0.009
Respondent has to queue to use toilet at night	22.91	91.27	8.73	χ^2^ (1, *N =* 549) = 6.36, *p =* 0.030
Respondent's toilet is sometimes not accessible during the day	77.64	85.48	14.52	χ^2^ (1, *N =* 549) = 2.51, *p =* 0.144
Respondent's toilet is sometimes not accessible at night	31.45	89.6	10.4	χ^2^ (1, *N =* 549) = 2.08, *p =* 0.180
Respondent pays fee to access daytime toilet	42.91	82.63	17.37	χ^2^ (1, *N =* 549) = 8.19, *p =* 0.017
Respondent pays fee to access nighttime toilet	3.82	90.48	9.52	χ^2^ (1, *N =* 549) = 0.21, *p =* 0.655
Walk time to reach daytime toilet				χ^2^ (4, *N =* 546) = 0.64, *p =* 0.531
Does not walk	31.09	88.89	11.11	
< 1 min	15.64	90.7	9.3	
Between 1 and 2 min	19.45	85.05	14.95	
Between 3 and 4 min	14.73	87.65	12.35	
Five or more min	19.09	80.95	19.05	
Walk time to reach nighttime toilet				χ^2^ (4, *N =* 546) = 2.82, *p =* 0.0805
Does not walk	66.18	85.16	14.84	
< 1 min	16.18	93.26	6.74	
Between 1 and 2 min	6.73	91.89	8.11	
Between 3 and 4 min	5.27	72.41	27.59	
Five or more min	5.64	93.55	6.45	
Respondent uses shared toilet during the day	79.82	85.65	14.35	χ^2^ (1, *N =* 549) = 1.79, *p =* 0.211
Respondent uses shared toilet at night	32.18	89.83	10.17	χ^2^ (1, *N =* 549) = 4.93, *p =* 0.051
Respondent uses public toilet during the day	50.36	81.59	18.41	χ^2^ (1, *N =* 549) = 25.95, *p =* 0.001
Respondent uses public toilet at night	7.45	78.05	21.95	χ^2^ (1, *N =* 549) = 6.45, *p =* 0.029
Respondent uses bags/buckets during the day	29.64	82.82	17.18	χ^2^ (1, *N =* 549) = 0.90, *p =* 0.364
Respondent uses bags/buckets at night	68.73	85.98	14.02	χ^2^ (1, *N =* 549) = 0.20, *p =* 0.662
Respondent's daytime toilet is clean	61.64	91.74	8.26	χ^2^ (1, *N =* 549) = 50.50, *p =* 0.000
Respondent's nighttime toilet is clean	29.09	91.25	8.75	χ^2^ (1, *N =* 549) = 5.75, *p =* 0.038
Respondent's daytime toilet has running water	32.12	92.05	7.95	χ^2^ (1, *N =* 549) = 7.62, *p =* 0.020
Respondent's nighttime toilet has running water	10.18	94.64	5.36	χ^2^ (1, *N =* 549) = 4.41, *p =* 0.062
Respondent's daytime toilet has a bin for pads	18.91	89.42	10.58	χ^2^ (1, *N =* 549) = 0.472, *p =* 0.508
Respondent's nighttime toilet has a bin for pads	7.64	90.48	9.52	χ^2^ (1, *N =* 549) = 0.36, *p =* 0.559
**Community/neighborhood-level factors**				
Primary water source				χ^2^ (3, *N =* 547) = 1.01, *p =* 0.390
Tap/well inside house/plot/building	13.45	86.49	13.51	
Tap/well outside house/plot/building	16.18	80.9	19.1	
Public tap/well	56.73	87.18	12.82	
Tanker/vendor	13.64	92	8	
Residential stability in community				χ^2^ (4, *N =* 546) = 1.8381, *p =* 0.185
< 1 year	5.27	86.21	13.79	
Between 1 and 4 years	24.91	89.05	10.95	
Between 5 and 9 years	24.00	91.67	8.33	
Between 10 and 19 years	2.00	80.91	19.09	
>20 years	25.82	84.51	15.49	
Neighborhood cohesion (mean)	0.78 (0.030)		100	OR = 0.43 (95% CI: 0.065, 2.85), *p* = 0.345
Neighborhood cohesion (sum)	14.10 (0.540)		100	OR = 0.95 (95% CI: 0.859, 1.060), *p* = 0.345
Neighborhood disorganization (mean)	2.28 (0.094)		100	OR = 3.96 (95% CI: 0.905, 17.291), *p* = 0.064
Neighborhood disorganization (sum)	36.44 (1.503)		100	OR = 1.09 (95% CI: 0.994, 1.195), *p* = 0.064
**Societal/cultural-level factors**				
Respondent's religion has rules about disposal of feces	8.18	75.56	24.44	χ^2^ (1, *N =* 549) = 4.41, *p =* 0.062
Respondent's religion has rules about hygiene	10.55	70.69	29.31	χ^2^ (1, *N =* 549) = 20.89, *p =* 0.001
Respondent's culture has rules about disposal of feces	19.82	81.65	18.35	χ^2^ (1, *N =* 549) = 0.8485, *p =* 0.378
Respondent's culture has rules about hygiene	18.73	79.61	20.39	χ^2^ (1, *N =* 549) = 1.93, *p =* 0.195

**Table 3:** Proportions and bivariate associations with past-year heard about sanitation-related violence.

	**% [mean (std)]**	**No viol**	**Yes viol**	**Chi-squared**
**Dependent variable**				
Heard about sanitation-related violence in past 12 months	23.64			–
**Individual-level factors**				
Respondent age (continuous)	32.21 (0.786)			OR = 1.01 (95% CI: 0.973, 1.052), *p* = 0.531
Respondent education level				χ^2^ (3, *N =* 547) = 0.551, *p =* 0.603
Less than primary	18.55	71.57	28.43	
Completed primary, no secondary	24.73	76.47	23.53	
Some secondary, but did not complete	22.18	77.05	22.95	
Completed secondary	34.55	78.42	21.58	
Respondent employed	45.45	74.8	25.2	χ^2^ (1, *N =* 549) = 0.305, *p =* 0.593
Respondent reports good/excellent health	57.82	73.9	26.1	χ^2^ (1, *N =* 549) = 0.712, *p =* 0.418
Respondent believes it is safe to walk alone in the neighborhood during the day	97.82	76.39	23.61	χ^2^ (1, *N =* 549) = 0.0110, *p =* 0.918
Respondent believes it is safe to walk alone in the neighborhood at night	19.64	87.96	12.04	χ^2^ (1, *N =* 549) = 3.568, *p =* 0.088
Respondent fears victimization during the day	0.91	60	40	χ^2^ (1, *N =* 549) = 0.859, *p =* 0.376
Respondent fears victimization at night	75.45	72.05	27.95	χ^2^ (1, *N =* 549) = 5.835, *p =* 0.036
Respondent identified insecurity as an issue with their toilet during the day	5.27	75.86	24.14	χ^2^ (1, *N =* 549) = 0.003, *p =* 0.961
Respondent identified insecurity as an issue with their toilet during the night	34.36	75.66	24.34	χ^2^ (1, *N =* 549) = 0.011, *p =* 0.919
Worried about crime in neighborhood				χ^2^ (2, *N =* 548) = 0.010, *p =* 0.986
Not at all worried	11.45	77.78	22.22	
A little worried	22.00	76.03	23.97	
Very worried	66.55	76.23	23.77	
Respondent is satisfied with their toilet during the day	32.91	75.69	24.31	χ^2^ (1, *N =* 549) = 0.073, *p =* 0.793
Respondent is satisfied with their toilet at night	50.91	73.21	26.79	χ^2^ (1, *N =* 549) = 2.91, *p =* 0.119
Respondent reports privacy in toilet during the day	67.09	81.3	18.7	χ^2^ (1, *N =* 549) = 4.75, *p =* 0.054
Respondent reports privacy in toilet at night	41.09	85.84	14.16	χ^2^ (1, *N =* 549) = 9.381, *p =* 0.012
Respondent is embarrassed to use toilet during the day	34.55	66.32	33.68	χ^2^ (1, *N =* 549) = 5.605, *p =* 0.039
Respondent is embarrassed to use toilet during the night	53.27	68.94	31.06	χ^2^ (1, *N =* 549) = 5.963, *p =* 0.035
Household monthly income (KES)				χ^2^ (2, *N =* 548) = 0.067, *p =* 0.931
Less than 5,000	25.09	78.26	21.74	
KES 5,000-10,000	50.55	75.54	24.46	
More than 10,000	24.36	76.12	23.88	
Relationship status				χ^2^ (3, *N =* 547) = 0.058, *p =* 0.976
Married	54.73	76.08	23.92	
Living with partner, but not married	2.00	72.73	27.27	
Regular partner, living apart	6.36	76.09	23.91	
Single	34.91	77.08	22.92	
Residential stability in household				χ^2^ (4, *N =* 546) = 1.192, *p =* 0.329
< 1 year	13.82	67.11	32.89	
Between 1 and 4 years	44.91	76.92	23.08	
Between 5 and 9 years	24.36	82.84	17.16	
Between 10 and 19 years	8.00	72.73	27.27	
>20 years	8.91	73.47	26.53	
Respondent has children	81.64	75.95	24.05	χ^2^ (1, *N =* 549) = 0.196, *p =* 0.668
Respondent number of children (continuous)	2.21 (0.067)			OR = 1.07 (95% CI: 0.855, 1.329), *p* = 0.536
Head of household is male	57.45	77.22	22.78	χ^2^ (1, *N =* 549) = 0.285, *p =* 0.605
Respondent participates in one or more groups	58.55	69.25	30.75	χ^2^ (1, *N =* 549) = 10.801, *p =* 0.008
**Technological-level factors**				
Respondent's daytime toilet has doors	85.09	73.5	26.5	χ^2^ (1, *N =* 549) = 10.74, *p =* 0.008
Respondent's nighttime toilet has doors	35.27	80.41	19.59	χ^2^ (1, *N =* 549) = 2.01, *p =* 0.187
Respondent's daytime toilet has locks on doors	74.91	75.49	24.51	χ^2^ (1, *N =* 549) = 0.170, *p =* 0.689
Respondent's nighttime toilet has locks on doors	32.91	81.77	18.23	χ^2^ (1, *N =* 549) = 3.626, *p =* 0.086
Respondent's daytime toilet has separate gender stalls	27.45	77.48	22.52	χ^2^ (1, *N =* 549) = 0.024, *p =* 0.880
Respondent's nighttime toilet has separate gender stalls	6.73	83.78	16.22	χ^2^ (1, *N =* 549) = 0.498, *p =* 0.497
Respondent's daytime toilet has lights	30.00	76.97	23.03	χ^2^ (1, *N =* 549) = 0.008, *p =* 0.932
Respondent's nighttime toilet has lights	10.36	89.47	10.53	χ^2^ (1, *N =* 549) = 3.042, *p =* 0.112
Respondent's daytime toilet is closed at night	42.91	68.22	31.78	χ^2^ (1, *N =* 549) = 13.190, *p =* 0.005
Respondent has to queue to use toilet during the day	63.64	72.86	27.14	χ^2^ (1, *N =* 549) = 2.903, *p =* 0.119
Respondent has to queue to use toilet at night	22.91	81.75	18.25	χ^2^ (1, *N =* 549) = 3.212, *p =* 0.103
Respondent's toilet is sometimes not accessible during the day	77.64	72.13	27.87	χ^2^ (1, *N =* 549) = 31.999, *p =* 0.000
Respondent's toilet is sometimes not accessible at night	31.45	79.19	20.81	χ^2^ (1, *N =* 549) = 0.651, *p =* 0.439
Respondent pays fee to access daytime toilet	42.91	67.8	32.2	χ^2^ (1, *N =* 549) = 11.590, *p =* 0.007
Respondent pays fee to access nighttime toilet	3.82	76.19	23.81	χ^2^ (1, *N =* 549) = 0.000, *p =* 0.992
Walk time to reach daytime toilet				χ^2^ (4, *N =* 546) = 1.842, *p =* 0.175
Does not walk	31.09	82.46	17.54	
< 1 min	15.64	86.05	13.95	
Between 1 and 2 min	19.45	74.77	25.23	
Between 3 and 4 min	14.73	71.6	28.4	
Five or more min	19.09	63.81	36.19	
Walk time to reach nighttime toilet				χ^2^ (4, *N =* 546) = 1.337, *p =* 0.285
Does not walk	66.18	76.65	23.35	
< 1 min	16.18	84.27	15.73	
Between 1 and 2 min	6.73	70.27	29.73	
Between 3 and 4 min	5.27	58.62	41.38	
Five or more min	5.64	74.19	25.81	
Respondent uses shared toilet during the day	79.82	71.98	28.02	χ^2^ (1, *N =* 549) = 19.76, *p =* 0.001
Respondent uses shared toilet at night	32.18	78.53	21.47	χ^2^ (1, *N =* 549) = 0.411, *p =* 0.536
Respondent uses public toilet during the day	50.36	67.51	32.49	χ^2^ (1, *N =* 549) = 18.62, *p =* 0.002
Respondent uses public toilet at night	7.45	65.85	34.15	χ^2^ (1, *N =* 549) = 1.758, *p =* 0.214
Respondent uses bags/buckets during the day	29.64	79.14	20.86	χ^2^ (1, *N =* 549) = 0.157, *p =* 0.700
Respondent uses bags/buckets at night	68.73	75.93	24.07	χ^2^ (1, *N =* 549) = 0.028, *p =* 0.870
Respondent's daytime toilet is clean	61.64	78.76	21.24	χ^2^ (1, *N =* 549) = 1.013, *p =* 0.338
Respondent's nighttime toilet is clean	29.09	82.5	17.5	χ^2^ (1, *N =* 549) = 3.223, *p =* 0.103
Respondent's daytime toilet has running water	32.12	84.66	15.34	χ^2^ (1, *N =* 549) = 6.645, *p =* 0.028
Respondent's nighttime toilet has running water	10.18	96.43	3.57	χ^2^ (1, *N =* 549) = 19.86, *p =* 0.001
Respondent's daytime toilet has a bin for pads	18.91	75.96	24.04	χ^2^ (1, *N =* 549) = 0.002, *p =* 0.962
Respondent's nighttime toilet has a bin for pads	7.64	78.57	21.43	χ^2^ (1, *N =* 549) = 0.105, *p =* 0.753
Primary water source				χ^2^ (3, *N =* 547) = 2.284, *p =* 0.132
Tap/well inside house/plot/building	13.45	91.89	8.11	
Tap/well outside house/plot/building	16.18	70.79	29.21	
Public tap/well	56.73	73.72	26.28	
Tanker/vendor	13.64	78.67	21.33	
Residential stability in community				χ^2^ (4, *N =* 546) = 0.804, *p =* 0.489
< 1 year	5.27	89.66	10.34	
Between 1 and 4 years	24.91	75.91	24.09	
Between 5 and 9 years	24.00	76.52	23.48	
Between 10 and 19 years	2.00	78.18	21.82	
>20 years	25.82	72.54	27.46	
Neighborhood cohesion (mean)	0.78 (0.030)			OR = 4.92 (95% CI: 0.262, 92.15), *p* = 0.254
Neighborhood cohesion (sum)	14.10 (0.540)			OR = 1.09 (95% CI: 0.928, 1.286), *p* = 0.254
Neighborhood disorganization (mean)	2.28 (0.094)			OR = 16.02 (95% CI: 3.970, 64.663), *p* = 0.001
Neighborhood disorganization (sum)	36.44 (1.503)			OR = 1.19 (95% CI: 1.090, 1.298), *p* = 0.001
Respondent's religion has rules about disposal of feces	8.18	60	40	χ^2^ (1, *N =* 549) = 2.090, *p =* 0.179
Respondent's religion has rules about hygiene	10.55	62.07	37.93	χ^2^ (1, *N =* 549) = 1.496, *p =* 0.249
Respondent's culture has rules about disposal of feces	19.82	56.88	43.12	χ^2^ (1, *N =* 549) = 4.072, *p =* 0.071
Respondent's culture has rules about hygiene	18.73	53.4	46.6	χ^2^ (1, *N =* 549) = 5.342, *p =* 0.043

### Ethics approval

The study was approved by ethics committees at Rutgers, The State University of New Jersey and the National Commission on Science, Technology, and Innovation [permit number NACOSTI/P/15/7495/7482] in Nairobi, Kenya.

## Results

### Participant characteristics

Descriptive statistics are presented in Table 1. The average age of participants in this study was just over 32 (M = 32.2, SD = 0.79). About 19% of participants had less than a primary education, 24% finished primary school, but did not attend secondary school, and the remaining 67% had at least some secondary education with almost 35% having finished. Less than half of the sample (45%) were employed. More than half the participants were married (55%), with only 35% being single. Most women (51%) reported monthly household incomes between Ksh 5,000–10,000 (~US $50–100), with about one-quarter having household incomes below 5,000 and one-quarter with incomes above 10,000. Over 94% of respondents reported living in Mathare for more than one year, with over one-quarter living in Mathare for more than 20 years. Over 86% of respondents also reported having lived in their current household for >1 year. Well over half (57%) reported good or excellent health.

Approximately 24% of the women in this study reported hearing about another woman experiencing violence when walking to or using a toilet or alternative site for urination/defecation in the past 12 months. Over 13% reported observing sanitation-related violence when accessing their primary sanitation site in the past year, and almost 13% reported past-year sanitation-related violence.

Most participants used more than one toilet or sanitation method throughout a 24-h period—sometimes using up to four different methods. During the day, approximately 31% of participants reported using a public toilet with another 28% using bags or buckets at home for catching feces/urine. About 16% used a shared toilet in their housing plot or building, and 12% used a privately-owned toilet shared with one or more other families. About 6% of participants reported using a private toilet and 7% reported using open defecation. At night, over 67% of women reported using bags or buckets in the home, which were then emptied/dumped into a system of open drainage ditches throughout the settlement. Only 12% of women reported using a shared toilet in their housing plot or building at night, 6% reported using a privately-owned shared toilet, 6% reported using open defecation, 5% reported using a public toilet, and 4% reported using a private toilet.

### Bivariate results

At the individual-level, *hearing about sanitation-related violence* was associated with fearing victimization at night [χ^2^ (1, *N* = 549) = 5.84, *p* = 0.036], being embarrassed to use daytime [χ^2^ (1, *N* = 549) = 5.61, *p* = 0.039] and nighttime [χ^2^ (1, *N* = 549) = 5.96, *p* = 0.035] toilets, and privacy in nighttime toilet [χ^2^ (1, *N* = 549) = 9.38, *p* = 0.012]. At the interpersonal-level, participation in one or more social groups was associated with past-year sanitation-related violence [χ^2^ (1, *N* = 549) = 10.80, *p* = 0.008]. Several technological-level factors or facility/site characteristics were associated with past-year sanitation-related violence, specifically respondents' daytime toilet/site having a door [χ^2^ (1, *N* = 549) = 10.74, *p* = 0.008], being closed at night [χ^2^ (1, *N* = 549) = 13.19, *p* = 0.005], being sometimes inaccessible [χ^2^ (1, *N* = 549) = 32.00, *p* = 0.000], being shared [χ^2^ (1, *N* = 549) = 19.76, *p* = 0.00], being public [χ^2^ (1, *N* = 549) = 18.62, *p* = 0.002], having running water [χ^2^ (1, *N* = 549) = 6.65, *p* = 0.028], and having to pay a fee to use it [χ^2^ (1, *N* = 549) = 11.59, *p* = 0.007]. At the community-level, a single-point increase in the mean of the perceived neighborhood disorganization scale was associated with 19% greater odds of hearing about past-year sanitation-related violence [OR = 1.19, 95% CI (1.090, 1.298), *p* = 0.001].

At the individual-level, a single-year increase in age was associated with 5% greater odds of having *observed sanitation-related violence* of another person in the past year. Additionally, past-year observed sanitation-related violence was associated with higher proportions of respondents fearing victimization [χ^2^ (1, *N* = 549) = 19.36, *p* = 0.001] and lower proportions of respondents reporting it is safe to walk alone at night [χ^2^ (1, *N* = 549) = 12.12, *p* = 0.006]. Observing past-year sanitation-related violence was also associated with being embarrassed to use one's daytime toilet [χ^2^ (1, *N* = 549) = 6.90, *p* = 0.025]. At the interpersonal/household-level, having children was associated with observing past-year sanitation-related violence [χ^2^ (1, *N* = 549) = 6.88, *p* = 0.026], with each additional child being associated with 20% higher odds of observing past-year sanitation-related violence [OR = 1.21, 95% CI [1.080, 1.354], *p* = 0.004]. Technological-level factors or facility/site characteristics, such as having to queue to use one's daytime [χ^2^ (1, *N* = 549) = 10.25, *p* = 0.009] and nighttime [χ^2^ (1, *N* = 549) = 6.36, *p* = 0.030] toilets and having to pay a fee to use one's daytime toilet [χ^2^ (1, *N* = 549) = 8.19, *p* = 0.017], were associated with observing past-year sanitation-related violence. Using a public toilet during the day [χ^2^ (1, *N* = 549) = 125.95, *p* = 0.001] and night [χ^2^ (1, *N* = 549) = 6.45, *p* = 0.029] were also associated with observing past-year sanitation-related violence. Lower proportions of respondents who reported that their daytime [χ^2^ (1, *N* = 549) = 50.50, *p* = 0.000] or nighttime [χ^2^ (1, *N* = 549) = 5.75, *p* = 0.038] toilets/sites/methods reported observing past-year sanitation-related violence. Finally, using a toilet with running water was associated with observing past-year sanitation-related violence [χ^2^ (1, *N* = 549) = 7.62, *p* = 0.020]. No community/neighborhood-level factors were significantly associated with observing past-year violence, but, at the social/cultural-level, belonging to a religion that has specific rules about hygiene was associated with observing past-year sanitation-related violence [χ^2^ (1, *N* = 549) = 20.89, *p* = 0.001].

Findings from bivariate tests of association, suggest that women's *experience of past-year sanitation-related violence* was significantly associated with hearing about [χ^2^ (1, *N* = 549) = 6.28, *p* = 0.031] and observing [χ^2^ (1, *N* = 549) = 12.33, *p* = 0.006] past-year sanitation-related violence. A higher proportion of women who experienced past-year sanitation-related violence reported observing and hearing about other women's past-year sanitation-related violence. At the individual-level, a single-year increase in age was associated with 4% greater odds of having experienced sanitation-related violence in the past year [OR = 1.04, 95% CI [1.015, 1.066], *p* = 0.005]. A larger proportion of women who reported being employed also reported experiencing sanitation-related violence in the past year [χ^2^ (1, *N* = 549) = 5.43, *p* = 0.042]. Fewer respondents who reported privacy in their daytime toilet reported having experienced past-year sanitation-related violence [χ^2^ (1, *N* = 549) = 4.00, *p* = 0.034]. Fewer respondents who reported that their nighttime toilets had locks on the doors [χ^2^ (1, *N* = 549) = 6.62, *p* = 0.028] or that their daytime [χ^2^ (1, *N* = 549) = 17.38, *p* = 0.002] or nighttime [χ^2^ (1, *N* = 549) = 7.22, *p* = 0.023] toilets were clean also reported experiencing past-year sanitation-related violence. At the community/neighborhood-level, an increase in perceived neighborhood cohesion was associated with lower odds of experiencing past-year sanitation-related violence [OR =0.17, 95% CI (0.033, 0.839), *p* = 0.033].

### Multivariate results

**Figure 1:**
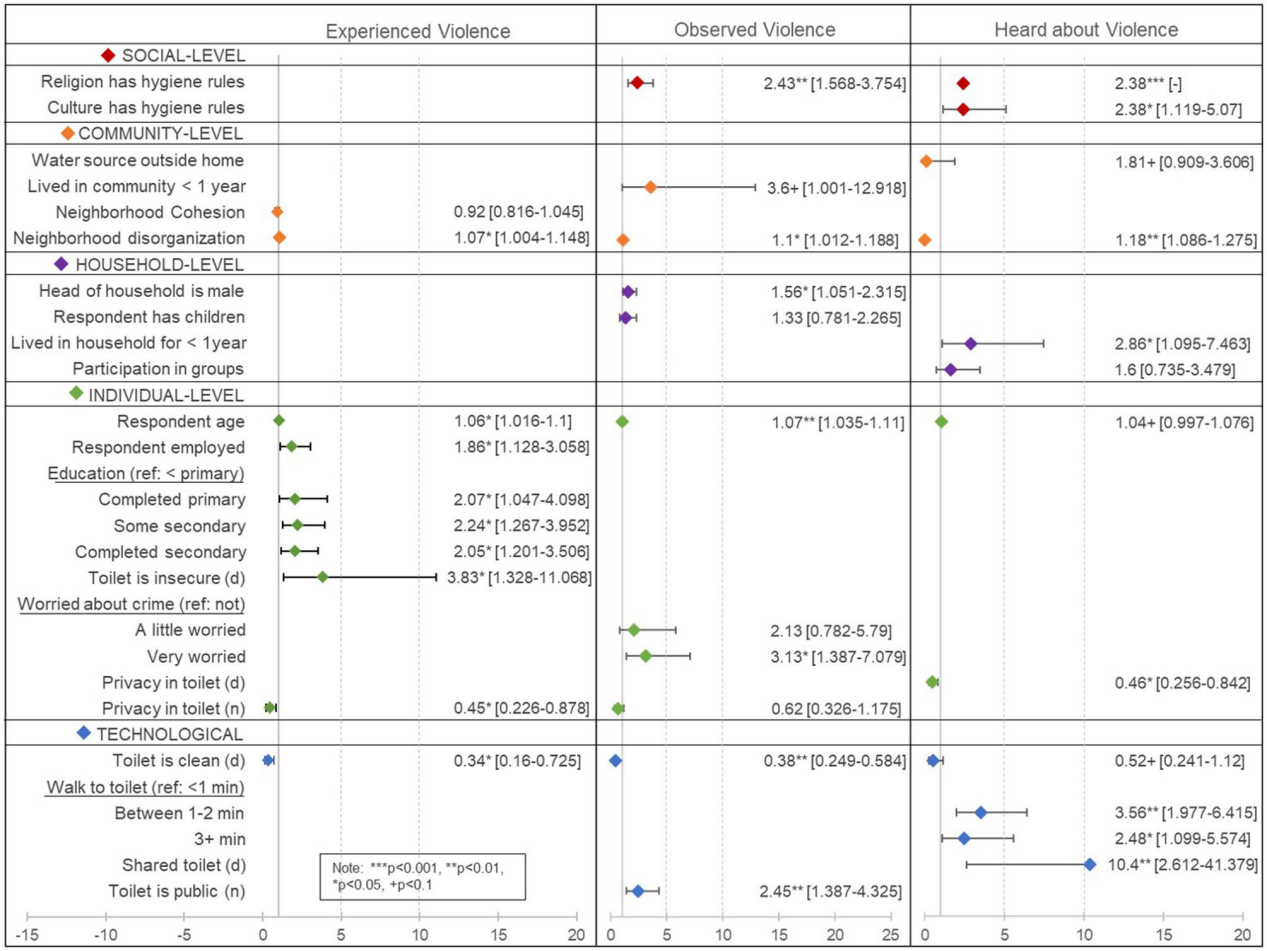
Results from multivariate logistic regressions of factors associated with having experienced, observed, and heard about past-year sanitation-related violence.

Results are summarized in Figure 1. Only one individual-level factor was significantly associated with *hearing about sanitation-related violence in the past year* in the multivariate logistic regression: having privacy in one's nighttime toilet/sanitation site, which was associated with 54% lower odds of hearing about sanitation-related violence [OR = 0.46, 95% CI (0.256, 0.842), *p* = 0.017]. At the household/interpersonal level, residential instability in one's house, i.e., living in one's house for <1 year was associated with almost three times the odds of hearing about past-year sanitation-related violence [OR = 2.86, 95% CI (1.095, 7.463), *p* = 0.035]. Technological factors such as using a shared toilet during the day [OR =0.17, 95% CI (0.033, 0.839), *p* = 0.033] and having to walk >1 min to reach one's toilet/site [OR_1 − 2minutes_ = 3.56, 95% CI (1.977, 6.415), *p* = 0.001; OR_3+minutes_ = 2.48, 95% CI (1.099, 0.839), *p* = 5.574] were associated with hearing about sanitation-related violence in the past-year. At the community/neighborhood-level, each single-point increase in the mean level of perceived neighborhood disorder was associated with higher odds of hearing about sanitation-related violence in the past year [OR = 1.18, 95% CI (1.089, 1.275), *p* = 0.033]. At the societal/cultural-level, belonging to a religion that has specific rules about hygiene was associated with more than double the odds of having heard about past-year violence [OR = 2.38, 95% CI (1.119, 5.070), *p* = 0.028]. AIC and BIC for the original and final, more parsimonious hearing about sanitation-related violence models were AIC_original_ = 481.8 and AIC_final_ = 459.1 and BIC_original_ = 705.7 and BIC_final_ = 510.8—suggesting improved model fit for the final model.

A single-year increase in age [OR = 1.07, 95% CI (1.035, 1.110), *p* = 0.001] and being very worried about crime in one's neighborhood (compared to not being worried at all) [OR = 3.13, 95% CI (1.387, 7.079), *p* = 0.011] were individual-level factors significantly associated with higher odds of having *observed past-year sanitation-related violence*. At the household-level, having a male head-of-household was associated with higher odds of having observed sanitation-related violence [OR = 1.56, 95% CI (1.051, 2.315), *p* = 0.031]. Only two technological factors or characteristics of one's toilet/sanitation site were associated with observing sanitation-related violence: reporting a clean daytime toilet/sanitation site was associated with lower odds of observing past-year sanitation-related violence [OR = 0.38, 95% CI (0.249, 0.584), *p* = 0.001] and using a public toilet at night was associated with more than double the odds of observing past-year sanitation-related violence [OR = 2.49, 95% CI (1.387, 4.325), *p* = 0.006]. At the community/neighborhood-level, having lived in the settlement for < 1 year was associated with more than three times the odds of observing past-year sanitation-related violence [OR = 3.60, 95% CI (1.001, 12.918), *p* = 0.05]. Additionally, increasing levels of perceived neighborhood disorganization were associated with higher odds of observing sanitation-related violence [OR = 1.10, 95% CI (1.012, 1.188), *p* = 0.028]. At the societal/cultural-level, belonging to a religion that has specific rules about hygiene was associated with more than double the odds of having observed past-year violence [OR = 2.43, 95% CI (1.568, 3.754), *p* = 0.001]. AIC and BIC for the original and final, more parsimonious observed sanitation-related violence models were AIC_original_ = 417.5 and AIC_final_ = 383.3 and BIC_original_ = 645.7 and BIC_final_ = 435.1. The lower values for AIC and BIC in the final model suggest improved model fit.

Several individual-level factors were associated with *experiencing past-year sanitation-related violence*. First, observing sanitation-related violence against another woman in the past-year was significantly associated with higher odds of also experiencing past-year sanitation-related violence [OR = 2.98, 95% CI (1.142, 7.786), *p* = 0.029]. A single-year increase in age [OR = 1.06, 95% CI (1.016, 1.100), *p* = 0.010], being employed [OR = 1.86, 95% CI (1.128, 3.058), *p* = 0.020], and increasing levels of education (compared to less than a primary education) [OR_primary_ = 2.07, 95% CI (1.047, 4.098), *p* = 0.039; OR_somesecondary_ = 2.24, 95% CI (1.267, 4.952), *p* = 0.010]; [OR_secondary_ = 2.05, 95% CI (1.201, 3.506), *p* = 0.014] were all associated with higher odds of having experienced past-year sanitation-related violence. Identifying insecurity as an issue with one's daytime toilet was associated almost four times the odds of having experienced past-year sanitation-related violence [OR = 3.83, 95% CI (1.328, 11.068), *p* = 0.018]. On the other hand, reporting privacy in one's nighttime toilet was associated with 55% lower odds of experiencing past-year sanitation-related violence [OR = 0.45, 95% CI (0.226, 0.878), *p* = 0.024]. Having access to a clean toilet during the day was also associated with lower odds of having experienced sanitation-related violence in the past year [OR = 0.34, 95% CI (0.160, 0.725), *p* = 0.010]. Finally, at the neighborhood/community-level, increasing levels of neighborhood disorganization were associated with higher odds of having experienced past-year sanitation-related violence [OR = 1.07, 95% CI (1.004, 1.148), *p* = 0.041]. Finally, AIC and BIC for the original and final expereinced sanitation-related violence models were AIC_original_ = 399.2 compared to AIC_final_ = 380.4 and BIC_original_ = 567.3 compared to BIC_final_ = 427.8.

## Discussion

The purpose of this study was to explore the prevalence and multilevel factors associated with women's past-year sanitation-related violence in Mathare informal settlement. We explored not only women's personal experiences of violence, but also the prevalence and factors associated with observing and hearing about other's experiences of sanitation-related violence in the community. Findings suggest that mostly individual- and technological-level factors were associated with experiencing past-year sanitation-related violence. Only one higher-level factor, neighborhood disorganization, was associated with experiences. On the other hand, factors significantly associated with observing and hearing about sanitation-related violence tended to be interpersonal/household-, community/neighborhood-, and social/cultural-level factors.

Individual-level factors, including participant's age, level of education, and employment status were associated with past-year experiences of sanitation-related violence. While quantitative explorations of sanitation-related violence are extremely limited, findings from a study focused on water, sanitation, and hygiene (WASH)-related violence and depressive symptoms in adolescent girls and young women in rural South Africa also found that age was associated with WASH-related violence (4) and in the same direction as our findings, with increasing age being associated with higher rates of reported violence. The direction of this association contradicts findings from general non-partner violence (NPV) literature, which suggest that odds of experiencing past-year NPV may decrease as age increases, but only in comparison to women between the ages of 15–19 years (11). Our study focused on women over the age of 18 years, which may account for the contradicting findings. Perhaps age has a non-linear relationship with general NPV and/or a different relationship specifically with sanitation-related violence that should be explored in future research.

Given the paucity of research focused on sanitation-related violence, it is difficult to speculate about the significance of the associations between other socio-demographic characteristics, such as participant level of education and employment and their experiences of sanitation-related violence. We looked to general NPV literature for suggestions. Increasing levels of education were found to be significantly associated with NPV in analyses focused on national datasets from several sub-Saharan African countries (11), which follows the same direction as our association. On the other hand, employment was associated with lower odds of NPV in four out of twenty countries in the same analysis (11). This contradicts our finding that employment increases the risk of sanitation-related violence. While there is limited explanation for why these factors may be associated with increasing or decreasing odds of NPV, in general, and sanitation-related violence, specifically, it may have to do with economic or environmental factors in informal settlements. For example, women who are employed in settlements may rely on toilets/sanitation-sites outside their homes, buildings, and communities on a regular basis. In particular, many of the women in Mathare are employed as domestic workers in more affluent neighborhoods abutting the informal settlement. These participants may have to negotiate using employers' toilets (52), and/or may be harassed when trying to use these facilities—contributing to experiences of violence.

Findings from this study suggesting that toilet/sanitation-related technological factors are associated with participants' experiences of sanitation-related violence are easier to interpret. Women in this study who identified that their toilets had privacy and were clean had lower odds of experiencing past-year sanitation-related violence. These findings are unsurprising given the evidence from other studies that suggest perceptions of privacy and cleanliness of toilet facilities are associated with women's and girls' perceptions of danger and risk (3, 53) and their experiences of sexual violence (2, 33). Findings from our study focused on hearing about and observing sanitation-related violence further highlight the importance of privacy and cleanliness of toilets for women's safety, i.e., analyses showed that hearing about sanitation-related violence was associated with limited privacy of one's toilet and observing sanitation-related violence was associated with unclean toilets. These findings highlight the critical importance of access to private and clean toilets, not only for public health, but for women's safety.

Women who specifically identified insecurity as an issue with their daytime toilet, also had almost four times the odds of having experienced past-year sanitation-related violence. Relatedly, women who reported that they were very worried about crime in their neighborhood, more generally, had more than three times the odds of observing sanitation-related violence in the past year. While these findings may seem self-explanatory, we found it interesting because fear of crime literature states that fear of victimization and actual victimization may not be correlated (54)—a global phenomenon known as the “paradox of fear” (55, 56). In this study, a perception of insecurity at toilets/sanitation sites or general worry about crime in the neighborhood are seemingly critical factors associated with actual experiences and observations of sanitation-related violence, respectively. Given the cross-sectional nature of the data, it is impossible to know whether identifying one's toilet as insecure or being worried about crime in one's neighborhood is associated with subsequent experiences or observations of violence or if experiences/observations of violence are associated with subsequent worry about toilet/sanitation or neighborhood insecurity. It seems likely that a woman who has experienced/observed sanitation-related violence at her primary toilet/sanitation site in the past year would subsequently report that her toilet/sanitation site and, potentially her neighborhood at large, are insecure, which may account for the strong correlation between these factors. But we want to also explore the possibility that a woman may identify that her toilet/sanitation site and/or neighborhood are insecure, but still “choose” to go to/utilize her primary toilet/sanitation site, thereby increasing her risk of sanitation-related violence.

According to fear of victimization theory, a person fearing victimization will often adopt defensive or avoidant behaviors to minimize their risk of actually experiencing violence (44, 57). We know, for example, that many women in informal settlements do this by reverting to using bags or buckets at home to capture feces and urine when (1) it is deemed unsafe to travel outside the home, building, or housing plot to use a toilet/sanitation site at night (58, 59) or (2) when women are menstruating and fear being verbally harassed at shared or public toilet facilities (60). Despite women's best efforts to minimize their exposure to violence, sanitation-related violence may violate some of the assumptions of fear of victimization theory, specifically that women will adopt avoidance or defensive behaviors like “choosing” not to visit a toilet/site outside their homes to minimize their risk.

There are several forms of violence for which women can adopt strategies to minimize exposure, such as avoiding crime hotspots in a neighborhood or staying inside at night. Adopting avoidance or defensive behaviors to minimize risk of sanitation-related violence, however, presents a dilemma. First, women cannot simply avoid meeting their sanitation needs all together; it is a requirement of life. Second, adopting avoidance strategies, e.g., not using a toilet, often places women and other members of the community at increased risk of sanitation-related infectious diseases, and, consequently, can open women up to stigma and harassment about their poor sanitation and hygiene practices. To be clear, adoption of avoidance and defensive behaviors, no matter the form of violence women are trying to minimize, has negative consequences. For example, avoidance strategies can increase walking times to reach a toilet/sanitation site or prevent individuals from engaging in work opportunities and social activities, as well other forms of community engagement (61). Worry about or fear of victimization is also associated with physiological effects including increased heart rate and rapid breathing (62); poorer overall health (63); and increased anxiety, depression, and post-traumatic stress disorder, helplessness and an overall decrease in quality of life (64, 65). But, for women trying to minimize their exposure to sanitation-related violence, the personal and collective consequences may be even more serious. For example, women who opt not to use a toilet and, instead, opt to use a bag or bucket in the home and dump the contents of these materials in nearby open drainages or opt to use open defecation nearby their homes, may be increasing theirs and their community's exposure to raw human waste and the potential negative health impacts of that. Additionally, they may face the stigma from community or outside members because of their “choices” not to utilize improved sanitation options and for putting others at risk. This may be why many women who report using bags and buckets in the home wake up very early in the morning when it is still dark to empty the contents into nearby drainages without being seen (60). This is one of the reasons sanitation-related violence is so critical to understand and prevent, and why strategies, such as the Sustainable Development Goal 6.2 to “ensure availability and sustainable management of water and sanitation for all” (66), truly pay attention to women's sanitation-related security.

Only one community-level factor, perceptions of neighborhood disorganization, was significantly associated with higher odds of past-year experiences of sanitation-related violence, but this variable was also associated with higher odds of hearing about and observing sanitation-related violence. Because of the cross-sectional nature of the data, it is possible that women heard about, witnessed, or experienced sanitation-related violence, which subsequently increased their perceptions of their neighborhoods as unsafe and disorganized. Alternatively, women who live in neighborhoods that they perceive as disorganized may be more likely to hear about, observe, or experience violence, particularly in higher-risk spaces such as public or shared toilets. Findings from this study, for example, suggest that women who rely on public toilets to meet their sanitation needs, particularly at night, have higher odds of having observed past-year sanitation-related violence. This corroborates findings from many studies that have identified public or shared toilets in informal settlements as potential “hotspots” for crime, especially sexual assault (1, 2, 4, 9, 67–69). In addition to sexual assault, toilets may also be sites of robbery, verbal abuse, or harassment, particularly at night (9), during times of tribal tensions (e.g., in periods leading up to presidential elections in Kenya) (58), and/or during menstruation (2, 60).

Several additional social/cultural-, community/neighborhood- and interpersonal/household-level factors were associated with hearing about and/or observing past-year sanitation-related violence. Increasing levels of neighborhood cohesion, for example, were associated with higher odds of hearing about and observing past-year sanitation-related violence. Being part of one or more social groups was also associated with higher odds of hearing about sanitation-related violence in the past year in bivariate analyses. These findings suggest that women's level of engagement in and perceptions of the social environment in informal settlements likely influences their opportunities for hearing about and potentially observing all sorts of community goings-on, including sanitation-related violence.

Relatedly, hearing about past-year sanitation-related violence was associated with longer walk times to toilets and with using a shared toilet during the day. It is possible that women who rely on toilets/sanitation sites outside their homes, buildings, or housing plots may accompany one another to these facilities, which is well-documented in other studies (7, 70), and they may share stories related to sanitation, including violence. Discussions from other literature focused on women's health in informal settlements in Kenya suggest that water taps outside the home may be spaces for women to come together, do laundry, fill jerry cans and socialize (71), and this may also be true of shared toilets, especially if women have to queue to use the facility. While public WASH-related spaces or open defecation are often considered “hotspots” for violence (1, 2, 4, 6, 72), as previously discussed, they may also provide spaces for women to share stories and discuss about critical goings-on that affect their security, for example sanitation-related violence.

Residential instability at the household-level (living in a household for <1 year) and at the community-level (living in the community for <1 year) were associated with higher odds of hearing about and observing sanitation-related violence in the past-year, respectively. While this is a challenging finding to interpret, it may indicate that the year leading up to the study survey had a uniquely high prevalence rate for violence, in general, and sanitation-related violence, in particular. This may have been the case given that the survey was collected in the few months leading up to presidential elections in 2016. Political tribalism is often incited by politicians in the time leading up to presidential elections in Kenya to canvas for support in informal settlements (73); thus, violence and harassment related to tribalism can be more intense during this time. Alternatively, this finding could suggest that women who are new to an area or community may be more alert to reports of or actual perpetration of violence—a phenomenon in the fear of victimization literature known as (hyper)vigilance (56).

This phenomenon may also be related to findings in our study that suggest respondents who report that their culture or religion has specific rules about hygiene have higher odds of hearing about or observing sanitation-related violence. Studies have suggested that women who have religious or cultural beliefs about menstruation, particularly beliefs about menstruation and menstrual blood being shameful, report a fear of being embarrassed or harassed, especially when using public toilets (60). While few women in informal settlements reported having experienced harassment themselves, they did report having heard about or observed other women being harassed, especially at public toilets (60). These stories may increase women's hypervigilance.

### Limitations and reflections on research and researchers

Findings from this study help provide insights into factors associated with sanitation-related violence in informal settlements; however, the study had a number of limitations. First, the data are cross-sectional; thus, inferences cannot be made about the directionality of associations between specific factors and sanitation-related violence. Future longitudinal studies would be advantageous in exploring temporal relationships between these factors and sanitation-related violence as well as programs, interventions, and factors in women's physical and social environment that might help to minimize sanitation-related violence.

Second, we used sanitation-related violence measures that have never been validated or tested before in any population. There are limited options for measuring sanitation-related violence, and we believe this measure was appropriate for capturing women's sanitation-related violence, but it is hard to know if this measure is accurately or adequately capturing these experiences, observations, or stories. This highlights a need for more research focused on the most appropriate sanitation-related measures for this population and setting.

Because sanitation-related violence is a relatively rare event, we had to dichotomize several variables or collapse the number of response categories for others to ensure there was an adequate distribution of sanitation-related violence frequencies across all categories of the independent variables. Dichotomizing continuous, ordinal, or nominal variables and collapsing response categories of multi-category nominal variables can lead to a loss of information and power.

Finally, findings and our interpretations should be viewed in light of the positionality and approaches of the researchers, authors, and study team (74). The study design was developed and overseen by a foreign-born white woman and a Kenyan-born Black woman who had never lived in an informal settlement. Although both researchers lived in Nairobi before, during, and after data collection for this study, had carried out previous research in this informal settlement, and could speak both Swahili and English, they were none-the-less outsiders to the community. Researchers worked with a team of women who lived in Mathare who contributed to development, review, editing, data collection, and translation throughout the study. Authors include a foreign-born white woman and a Kenyan-born Black woman who were part of the original research team and a foreign-born white woman who is an expert in violence against women.

### Conclusion

Overall, findings from this study seem to suggest that social/community engagement, including the use of and walk-time to public or shared WASH-related spaces outside the home, and social/cultural beliefs are important considerations for hearing about and observing sanitation-related violence, but less so experiences of sanitation-related violence. Alternatively, individual-level and technological factors may be critical factors in actual experiences of violence. These findings are important because they suggest that technical aspects of a toilet, such as privacy, cleanliness, and an “environment of safety/security”, may be the most important factors for actually preventing sanitation-related violence for women in informal settlements, which is helpful information for sanitation-related development agendas and goals (e.g., Sustainable Development Goal 6.2). However, findings also suggest that social, community, and interpersonal level factors may be the most important factors in helping to build up or diminish that “environment of safety/security”, and, relatedly, women's use of toilets/sites for sanitation in the first place. For example, hearing about and observing sanitation-related violence are likely to contribute to women's perceptions of toilets as unsafe environments and may influence their decisions about whether or not to utilize those toilets/sanitation sites, especially at night. However, sanitation-related violence is, as we suggest, complicated. Unlike other forms of NPV, such as sexual harassment or robbery, that can often be avoided by adopting avoidance or defensive strategies such as avoiding certain crime “hotspots”, traveling in groups, or not going out of the house at night (not that any woman should have to adopt any of these behaviors to increase their chances of not being victimized!), these avoidance strategies in relation to sanitation may exacerbate other forms of violence for women. For example, using bags or buckets in the home and dumping them in nearby drainages or relying on open defecation near the home to stay safe at night can expose women to verbal harassment, stigma, and criticism for not following socially-acceptable hygiene protocols, for putting others at risk of sanitation-related infectious diseases, and for contributing to the degradation of the environment. Most women might put up with the latter form of violence rather than risk sexual, physical, and/or verbal harassment at a toilet/sanitation site, but this should not be a tradeoff that women have to grapple with. Thus, sanitation-related violence and creating an environment of safety in which women can take care of their sanitation-related needs in ways that also protect them, their families, and their communities is a critical development issue.

## Data availability statement

The raw data supporting the conclusions of this article will be made available by the authors, without undue reservation.

## Ethics statement

The study was approved by Ethics Committees at Rutgers, The State University of New Jersey and the National Commission on Science, Technology, and Innovation [NACOSTI/P/15/7495/7482] in Nairobi, Kenya. The studies were conducted in accordance with the local legislation and institutional requirements. The participants provided their written informed consent to participate in this study.

## Author contributions

SW managed the data collection and analyzed the data. She was a major contributor in writing the manuscript. LJ was a major contributor in writing the manuscript. MD assisted in the collection of data. She was a major contributor in writing the manuscript. All authors read and approved the final manuscript.
